# Mapping network connection among symptoms of anxiety, depression, and sleep disturbance in Chinese high school students

**DOI:** 10.3389/fpubh.2022.1015166

**Published:** 2022-09-23

**Authors:** Shujian Wang, Wenxin Hou, Yanqiang Tao, Zijuan Ma, Kai Li, Yanling Wang, Zhaoyuan Xu, Xiangping Liu, Liang Zhang

**Affiliations:** ^1^Faculty of Psychology, Beijing Normal University, Beijing, China; ^2^Beijing Key Laboratory of Applied Experimental Psychology, National Demonstration Center for Experimental Psychology Education, Beijing, China; ^3^School of Psychology, South China Normal University, Guangzhou, China; ^4^Bengbu Second Middle School, Bengbu, China; ^5^The First Psychiatric Hospital of Harbin, Harbin, China; ^6^College Students' Mental Health Education Center, Northeast Agricultural University, Harbin, China; ^7^College of Education for the Future, Beijing Normal University, Zhuhai, China

**Keywords:** depression, anxiety, sleep disturbance, high school students, network analysis

## Abstract

**Background:**

Due to tremendous academic pressure, Chinese high school students suffer from severe depression, anxiety, and sleep disturbances. Moreover, senior high school students commonly face more serious mental health problems than junior high school students. However, the co-occurrence and internal relationships of depression, anxiety, and sleep disturbances clusters are scarcely examined among high students. Therefore, the current study inspected relationships between depression, anxiety, and sleep disturbance symptoms through network analysis and identified key symptoms bolstering the correlation and intensifying the syndromes.

**Methods:**

A total of 13,999 junior high school students (*M*_age_ = 13.42 years, *SD*_age_ = 1.35, 50% females) and 12,550 senior high school students (*M*_age_ = 16.93 years, *SD*_age_ = 1.67, 47% females) were recruited in Harbin. We constructed networks for all students, junior high group, and senior high group, including data from the Youth Self-rating Insomnia Scale-3 (YSIS-3), the Generalized Anxiety Disorder-2 (GAD-2), and the Patient Health Questionnaire-2 (PHQ-2). The indices of “strength” was used to identify symptoms' centrality, and “bridge strength” was used to find specific nodes that could bridge anxiety, depression, and sleep disturbance.

**Results:**

The networks of all students, junior high and senior high students, were stable and accurate. Among all networks, “Nervousness” (GAD1) had the highest strength, and “Nervousness”–“Excessive worry” (GAD1-GAD2) had the strongest correlation. “Nervousness” (GAD1) also functioned as the bridge symptom among junior high students, while “Sad mood” (PHQ2) among senior high students. Senior high students scored higher than junior high students on all items and had a tighter network structure.

**Conclusions:**

In networks consisting of anxiety, depression, and sleep disturbance, anxiety plays a conspicuous role in comorbidity among junior high school students, which transforms into depression among senior high school students. Treatments or interventions should be focused on these critical symptoms.

## Introduction

By 2020, China's number of high school students (7–12th grade) has exceeded 120 million (1). According to the latest survey, the prevalence of depression and anxiety in Chinese high school students has been 7.1 and 12.8%, respectively (2). Depression and anxiety are caused by academic pressure or low academic achievement (3), which bring great harm to students (4), including fatigue (5) and even suicide (6). Additionally, during the pubertal period, high school students are vulnerable to depression and anxiety (7). Moreover, depression and anxiety in high school students without intervention are potent catalysts of anxiety and depression in adulthood (8).

For Chinese high school students, the main role of routine life is to study. However, academic pressure was proved to be a notorious factor related to anxiety and depression (9, 10). Though the Chinese government endeavors in the education revolution, high school students still need to study on campus for 8.6 h or even over 12 h per day (3). At the same time, Chinese teachers also tend to focus on students' academic performance rather than their mental health, which leads to exceptionally scarce psychological support from teachers in mainland China (11). Moreover, compared to American counterparts, Crystal (12) has found that Chinese students perceived more parental academic expectations and less parental satisfaction, which results in more depressive mood and somatic complaints. In confronting stress from a hostile societal environment, teachers, and parents, high school students' depression and anxiety moods are difficult to channel and de-escalate.

Besides academic stress, a tangible factor, the pubertal period, is key for interpersonal relationships (13). In an investigation done by Hernandez (14), loneliness and feeling unloved were critical in depression and anxiety. For Chinese high school students, heavy academic stress squeezes the time for peer relationship construction, which is important for development (13). Naturally, with heavy academic stress and without normal peer relationships, high school students are susceptible to depression and anxiety (15).

Besides depression and anxiety, which can negatively affect high school students, sleep disturbance is also a quotidian factor that can influence high school students' mental health. A meta-analysis (16) showed that 26% of Chinese high school students suffer from sleep disturbance, which is higher than university students (17) and adults (18). Furthermore, from a longitudinal study (19), the onset of sleep disturbance is highly associated with the later onset of poor mental health. Furthermore, there is a significant positive correlation between depression, anxiety, and sleep disturbance in high school students (20).

Numerous studies have demonstrated high comorbidity rates between depression and anxiety with sleep disturbances (21–23). In a longitudinal study, Roane and Taylor (24) found that adolescents with sleep disturbance are more likely to develop and maintain depression symptoms. Yet, the study did not distinguish insomnia symptoms from delayed sleep phase syndrome nor present specific sleep disturbance symptoms which can be associated with depression and anxiety. In another longitudinal study, scholars investigated into co-currency between sleep disturbance and depression (25). Though this previous study indicated a causal relationship between depression and sleep disturbance, researchers stayed on the disease dimension and ignored the effect of anxiety. As a matter of fact, sleep disturbance, anxiety, and depression are all clusters of multiple specific symptoms, such as difficulty maintaining sleep, anhedonia, and excessive worry. They dynamically interweave in both diagnosis and treatment. The change in any visible symptoms would inevitably cause changes in the whole picture. Studies from the traditional perspective only focused on the relationship between the overall profile of psychological disorders, such as Guo (20) used the total scores of the Chinese Pittsburgh Sleep Quality Index and Center for Epidemiology Scale for Depression to explore the correlates between sleep disturbance and depression among 7–12th grade Chinese students. Instead of a simple combination of symptoms, a student may trap in a worrying mood for waking up too early in the morning and then be difficult to initiate sleep during the night for the worrying mood throughout the day. These previous studies ignored the interrelations between specific symptoms and heterogeneity of symptoms (26, 27), underestimating the complexity of mental health issues (28).

The network approach is a novel approach that views mental health problems as a system where symptoms reinforce or inhibit each other (29). From the psychotherapeutic viewpoint, network analysis has more practical value because it can identify core symptoms (30) and reveal how symptom clusters are linked (31), which can help us understand the intricate symptom structure (27). Several researchers have applied a network approach to studies of anxiety, depression, and sleep disturbance co-morbidity in the elderly and college students (32, 33). In research of elderly adults *via* network analysis, “Nervousness” is the most obvious symptom, whereas in research of college students, “sleep dissatisfaction”, “poor sleep quality”, and uncontrollable worry' occupied most nodes' strength (32, 33). Admittedly, intervention and treatment of depression and anxiety of elderly and college students can be of great value. As a complex developmental phase (13), depression and anxiety has profound impacts on the psychological development of high school students who continuously encounter academic stress (9), poor peer relationship (14), and scarce support. Hence, early intervention and treatment can largely save human resources and costs (8). However, no studies have focused on the relationships between symptoms of anxiety, depression, and sleep disturbance in high school students, despite the severity of this problem in them.

Thus, the current study used a network approach to explore the interactions between each specific symptom of sleep disturbance, depression, and anxiety, targeting to indicate key issues in intervention and prevention. Besides, due to the heavier study burden, the severity of sleep disturbance, anxiety, and depression in senior high school students is higher than in junior high school students (34, 35). Therefore, we also compare the depression-anxiety-sleep disturbance network between junior and senior high school students to tailor recommendations for students in different stages.

## Method

### Participants

This study was conducted between November 2021 and March 2022 in 35 high schools in Harbin by convenient sampling. We used Wenjuanxing, an online questionnaire platform (https://www.wjx.cn), and collected 13,999 junior high school students (*M*_age_ = 13.42 years, *SD*_age_ = 1.35, 50% females) and 12,550 senior high school students (*M*_age_ = 16.93 years, *SD*_age_ = 1.67, 47% females) datasets. Students had to provide signed informed consent before participating in the assessment. Given the sensitive nature of some questions (such as suicidal ideation for another research program), professional clinicians will timely intervene. The research was examined and approved by the ethical of Beijing Normal University (Reference number: 202112220084).

### Measures

#### Patient health questionnaire (PHQ-2)

The Two-item Patient Health Questionnaire (PHQ-2) is a widely used scale for screening depression symptoms (36). Participants were asked about the frequency [not at all (0), several days (1), more than half of the days (2), nearly every day (3)] of experiencing given symptoms in the last 2 weeks, and higher scores indicate more severe depression symptoms. The Chinese version of PHQ-2 was proved to be valid and reliable (37). In the current study, PHQ-2 has a high internal consistency with Cronbach α values of 0.80 and 0.84 in junior and senior high school students.

#### Generalized anxiety disorder scale (GAD-2)

The Generalized Anxiety Disorder Scale (GAD-2) is a valid and reliable assessment to screen generalized anxiety symptoms (38). Participants answered two questions about the frequency of core anxiety symptoms that occurred over the last 2 weeks. Each item scored from 0 (not at all) to 3 (nearly every day), with a higher score indicating more severe anxiety symptoms. The Chinese version also has good psychometric properties for identifying anxiety (39). In the current study, GAD-2 has a high internal consistency with Cronbach α values of 0.87 and 0.90 in junior and senior high school students.

#### Youth self-rating insomnia scale (YSIS-3)

We selected three questions from Youth Self-rating Insomnia Scale (YSIS-3) (40), a 5-point Likert questionnaire assessing sleep disturbance in the last month. Participants answered three questions, “Difficulty initiating sleep,” “Difficulty maintaining sleep,” and “Early morning awakening,” scoring from 1 (Very Satisfied) to 5 (Very Unsatisfied). Total scores in this questionnaire range from 3 to 15, and higher scores indicate poorer sleep quality. Previous studies have shown that YSIS in Chinese is valid and reliable (32, 33). In the current study, YSIS-3 has a high internal consistency with Cronbach α values of 0.84 in both junior and senior high school students.

### Network analysis

#### Item check

We used R (version 4.12) (41) to conduct all analyses. *Means, standard deviations* (*SD*s), *skewness*, and *kurtosis* of all GAD-2, PHQ-2, and YSIS-3 item scores were checked by the R package *psych 2.0.12* (42). According to the previous study (43), items should be excluded if they were poorly informative (2.5 *SD* below the mean item *SD*).

#### Network estimation

An extended Bayesian information criterion (EBIC) graphical least absolute shrinkage and selection operator (LASSO) model (44) was used to estimate the network. Each node (i.e., item) in the network represents a symptom, and each edge is the correlation between two symptoms. All variables were treated as continuous variables. The correlation matrix was shrunk to obtain easier and sparser networks. Blue and red edges represent positive and negative correlations. The R packages *bootnet 1.4.3* and *qgraph 1.6.9* were used for network estimation and visualization (45, 46).

Strength centrality, closeness, and betweenness are three common centrality indices used to assess the network property. Since closeness and betweenness are argued to be unreliable in determining nodes' importance according to previous research (47), we use strength to assess the nodes' centrality in this study. The predictability (i.e., *R*^2^) was estimated by the R package *mgm 1.2-12* (48).

#### Network stability and accuracy

The case-dropping bootstrap procedure was used to assess the stability of centrality indices (46), providing the correlation stability coefficient (*CS-C*). The *CS-C* represented the most proportion of samples that could be removed, with a 95% probability that the correlation between the original centrality indices would be at least 0.70. Generally, the *CS-C* should be ≥0.25, preferably ≥0.5. Bootstrapped confidence intervals (95% *CI*s) were computed to analyze the accuracy of edges. The narrower *CI*s indicated a more accurate network (46). Differences between edge-weights and centrality strengths were also analyzed by bootstrap tests based on 0.95 *CIs*. If *CIs* did not include zero, there was a statistical difference between two edges or two nodes. All analyses above were performed by the R package *bootnet* (Version 1.4.3) (46).

#### Bridge symptoms

Bridge symptoms represent the channel between different disorders. Referring to previous research (49), we screened bridge symptoms on the criterion of standardized values of bridge strength ≥1 in the current research (50).

#### Network comparison (covariating sex)

The network comparison test (NCT) was used to assess the difference in edge invariance (distributions of edge weights) and global strength (sum of all edge weights) between the networks in junior and senior high school students by the R package NetworkComparisonTest (Version 2.2.1) (51). Since depression, anxiety, and sleep disturbance symptoms tend to change with sex (20), we added sex as the covariate in the network model and made a comparison with the original network.

## Result

### Descriptive statistics and item check

The *means, standard deviations, skewness, kurtosis, strength*, and *predictabilities* of symptoms were shown in Table 1. No items were poorly informative.

**Table 1:** Descriptive information of all students, junior and senior high school students.

	**Item**	***M***	***SD***	**Skewness**	**Kurtosis**	**Strength^a^**	**Predictability**
All (26549)	PHQ1	0.49	0.82	1.68	1.97	−1.14	0.20
	PHQ2	0.38	0.73	2.08	3.84	0.18	0.30
	GAD1	0.40	0.75	2.04	3.57	1.60	0.62
	GAD2	0.31	0.71	2.47	5.55	1.04	0.69
	YSIS3	1.54	1.02	1.95	2.97	−0.76	0.51
	YSIS4	1.37	0.85	2.61	6.49	−0.58	0.52
	YSIS5	1.34	0.85	2.84	7.73	−0.34	0.46
Junior (13999)	PHQ1	0.38	0.73	2.04	3.53	−1.32	0.44
	PHQ2	0.29	0.65	2.53	6.22	−0.10	0.53
	GAD1	0.28	0.63	2.57	6.60	1.69	0.32
	GAD2	0.21	0.59	3.13	9.95	0.89	0.49
	YSIS3	1.44	0.91	2.22	4.38	−0.58	0.35
	YSIS4	1.33	0.79	2.73	7.45	−0.51	0.51
	YSIS5	1.30	0.79	3.03	9.09	−0.07	0.41
Senior (12550)	PHQ1	0.62	0.90	1.36	0.88	−1.13	0.19
	PHQ2	0.48	0.80	1.72	2.27	0.39	0.20
	GAD1	0.53	0.85	1.62	1.75	1.45	0.09
	GAD2	0.43	0.82	1.98	3.02	1.11	0.21
	YSIS3	1.64	1.12	1.69	1.81	−0.89	0.04
	YSIS4	1.41	0.92	2.46	5.44	−0.51	0.16
	YSIS5	1.38	0.92	2.65	6.42	−0.43	0.13

a The value of node strength was standardized Z scores from the network.

### Network structure

**Figure 1:**
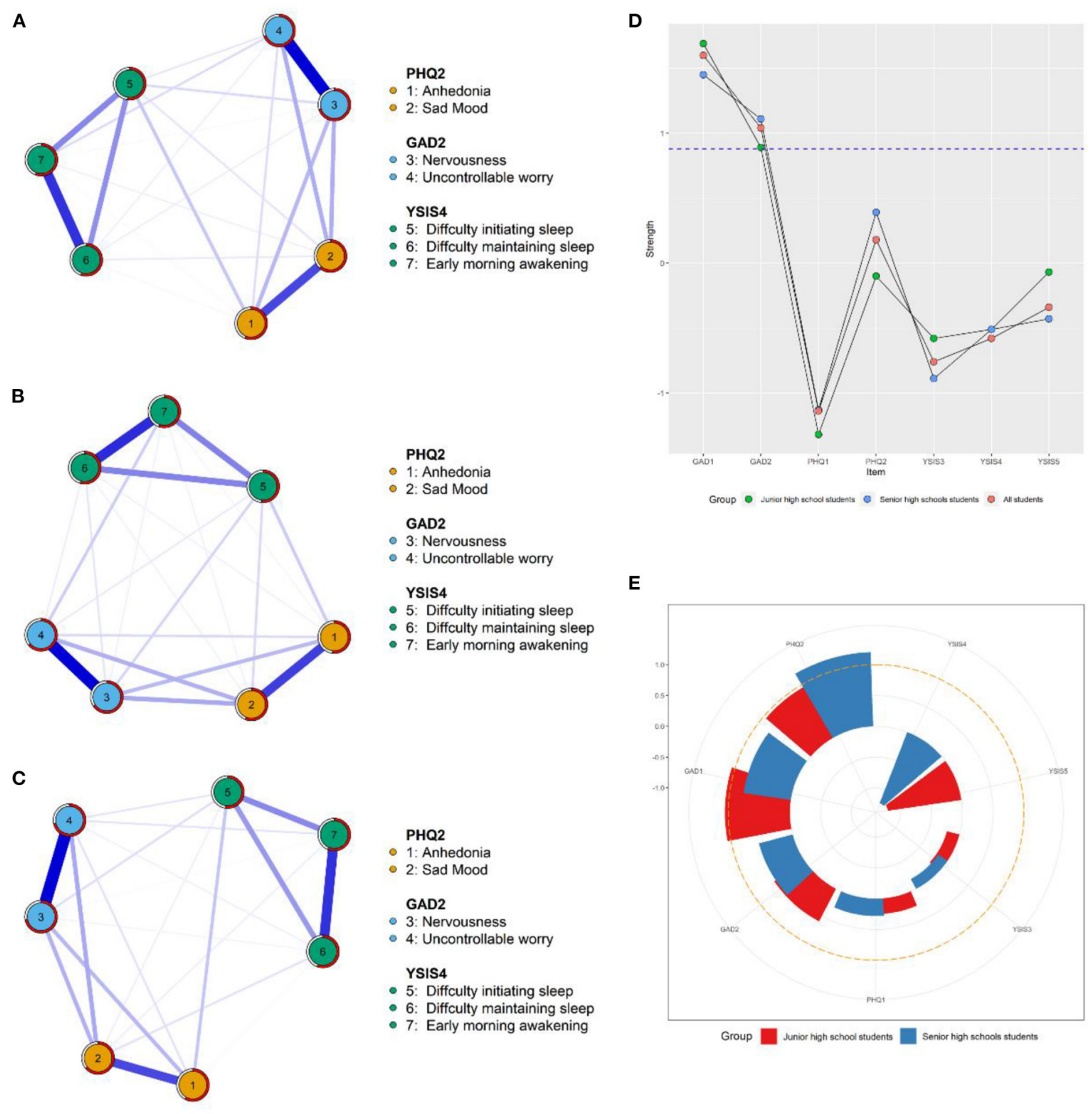
Network structures and centrality indexes. **(A)** Network of all students. **(B)** Network of junior high school students. **(C)** Network of senior high school students. **(D)** The strength centrality value among all students, junior high group, and senior high group. **(E)** The bridge centrality value among junior high group and senior high group.

Three depression-anxiety-sleep disturbance symptom networks were shown in Figure 1 and Supplementary Figure S1, and weighted adjacency matrixes were presented in Supplementary Tables S1–S3.

For all students, all edges (21) were not zero, and all these edges were positive. The edge of “Nervousness”–“Uncontrollable worry” (GAD1–GAD2) showed the strongest association, followed by the edge of “Difficulty maintaining sleep”–“Early morning awakening” (YSIS4–YSIS5) and the edge of “Anhedonia”–“Sad Mood” (PHQ1–PHQ2). In Table 1 and Figure 1D, “Nervousness” (GAD1) had the highest node strength among all students, followed by “Uncontrollable worry” (GAD2) and “Sad mood” (PHQ2), and an average of 60.2% of the variance could be potentially accounted by each node's neighbors (*M*_predictability_ = 0.60 ± 0.07). “Sad mood” (PHQ2) emerged as the bridge symptom among all students.

For junior high school students, all edges (21) were not zero, and all these edges were positive. The edge of “Nervousness”–“Uncontrollable worry” (GAD1–GAD2) showed the strongest association, followed by the edge of “Difficulty maintaining sleep”–“Early morning awakening” (YSIS4–YSIS5) and the edge of “Anhedonia”–“Sad Mood” (PHQ1–PHQ2). In Table 1 and Figure 1D, “Nervousness” (GAD1) had the highest node strength among junior high school students, followed by “Uncontrollable worry” (GAD2) and “Early morning awakening” (YSIS5), and an average of 57.1% of the variance could be potentially accounted by each node's neighbors (*M*_predictability_ = 0.57 ± 0.06). In Figure 1E, “Nervousness” (GAD1) emerged as the bridge symptom.

For senior high school students, 20 edges were not zero among 21 possible edges (95.2%), and all these edges were positive. The edge of “Nervousness”–“Uncontrollable worry” (GAD1–GAD2) showed the strongest association, followed by the edge of “Difficulty maintaining sleep”–“Early morning awakening” (YSIS4–YSIS5) and the edge of “Anhedonia”–“Sad Mood” (PHQ1–PHQ2). In Table 1 and Figure 1D, “Nervousness” (GAD1) had the highest node strength in the network among senior high school students, followed by “Uncontrollable worry” (GAD2) and “Sad mood” (PHQ2), and an average of 61.5% of the variance could be potentially accounted for by each node's neighbors (*M*_predictability_ = 0.62 ± 0.08). In Figure 1E, “Sad mood” (PHQ2) emerged as the bridge symptom.

### Network accuracy and stability

**Figure 2:**
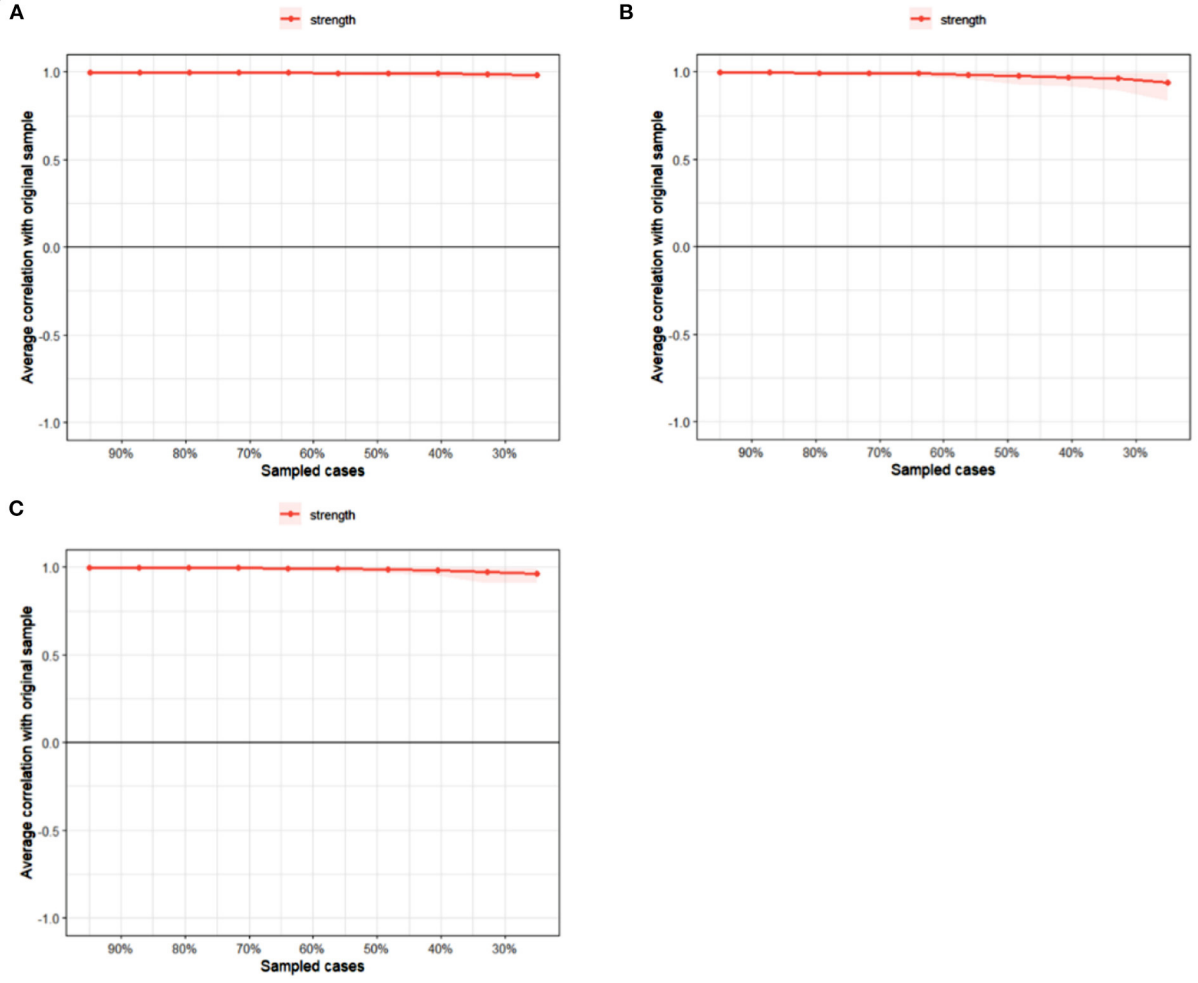
Case-dropping bootstrap test of strength centrality. The x-axis indicates the percentage of cases of the original sample included at each step. The y-axis indicates the correlations between the strength centrality from the original network and the strength centrality from the networks re-estimated after excluding increasing percentages of cases. **(A)** All students. **(B)** Junior high school students. **(C)** senior high school students.

In Figure 2, the case-dropping bootstrap procedure showed that *CS* coefficients were all above 0.75 in three groups, suggesting excellent stability for centrality indicators. 95% bootstrapped *CI*s of edges were narrow (Supplementary Figure S2), suggesting that edges were trustworthy. Additionally, results of the nonparametric bootstrap procedure revealed that most comparisons among edge weights and node strengths were statistically significant (Supplementary Figures S3, S4).

### Network comparison between junior and senior high school students

**Figure 3:**
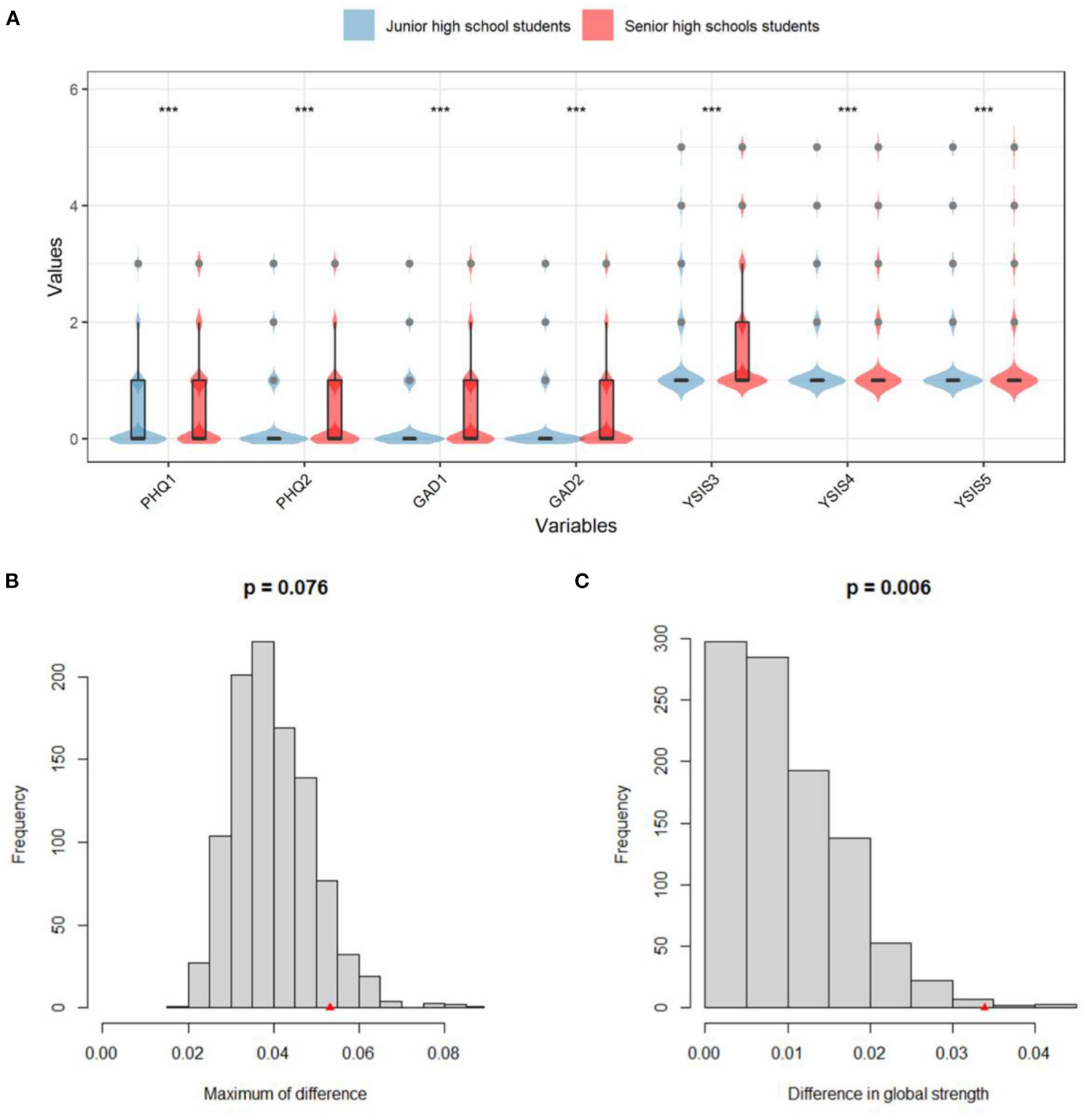
*T*-test results of all items and NCT results between junior and senior high school students. **(A)** *T*-test results of PHQ-2, GAD-2, and YSIS-3. **(B)** Network edge invariance. **(C)** Network global invariance.

The *t*-test result was shown in Figure 3A and Supplementary Table S4. All item scores in senior high group were significantly higher than junior high group (*p* < 0.001).

A permutation test was adapted to analyze invariance in different network characteristics. The results were shown in Figure 3. The value of the maximum difference in any edge weights (1,000 permutations) was not significant (*M* = 0.05, *p* = 0.08) (Figure 3B). The value of the difference in global network strength was significant (junior group = 3.12; senior group = 3.16, *p* < 0.01; Figure 3C).

The Spearman's correlations between the networks without covariate (sex) and with covariate in all three groups were all significant (*p* < 0.001), indicating that network structures did not change significantly between different genders.

### Sensitivity analyses

To test whether the results were influenced by sampling and a large sample size, we constructed new networks by randomly selecting 30% of the original sample and comparing them with the original networks.

The Spearman's correlation between the new network and the original network of all students, junior high students, and senior high students were 0.98 (*p* < 0.01), 0.97 (*p* < 0.01), and 0.96 (*p* < 0.01), respectively. The NCT results showed that between the new network and the original network in all students, junior high school students, and senior high school students, the value of the maximum difference in any edge weights and the difference in global network strength were all not significant (in Table 2 and Supplementary Figures S5–S7). The results revealed that network structures in the current study did not vary with the sample.

**Table 2:** The NCT results between the new network (30% sample size) and the original network.

	***M***	***p***	***S*_new_**	***S*_original_**	***p***
All students	0.02	0.99	3.16	3.15	0.73
Junior high students	0.03	0.99	3.11	3.12	0.63
Senior high students	0.04	0.84	3.16	3.16	0.98

*M* was the indicator of edge invariance, *S* was the indicator of global strength.

## Discussion

The current study explored the depression-anxiety-sleep disturbance symptom network structure among high school students and compared the network structure between junior and senior high school students. To the best of our knowledge, this was the first study using a network approach to analyze the relationship between depression, anxiety, and sleep disturbance among high school students. Some results are worth discussing.

In both junior and senior high school students, “Nervousness” (GAD1) had the highest central strength value, indicating that nervousness was the prominent symptom in the whole depression-anxiety-sleep disturbance symptom network. Meanwhile, “Nervousness” and “Uncontrollable worry” (GAD1–GAD2) had the strongest association. Uncontrollable worry is repetitive concerns or thoughts about potential adverse events or risks, and nervousness is an uneasy reaction to imminent disasters (52). The former is a negative feeling about a prolonged event, while the latter is about a temporary event. Both these two symptoms are anxiety disorder's main manifestations (38), which is consistent with previous researches (21, 23). Whilst, the results showed that concerns about short-term pressure events were also an influential factor in triggering sleep disturbances in high school students. As the most crucial examination in China, the national college entrance exam has received widespread attention from researchers, and a large number of studies have demonstrated the facilitating effect of the college entrance exam on Chinese high school students' anxiety (53, 54). However, in China's education system, known as “exam-oriented education,” students not only have to deal with the college entrance exam, but also with numerous quizzes (55). Most Chinese high school students are faced with monthly or even weekly tests. These relatively less crucial but frequent tests throw students into chronic nervousness, and have caused 45.9% of high school students to suffer from test anxiety (56). Students may be too nervous to maintain sleep because of frequent subtests. Besides, Xin and Yao (57) found that except for academic pressure, Chinese high school students also face many daily stressful events in school, such as interpersonal conflicts and fear of punishment from teachers. These worrying feelings and chronic nervousness play an important role in students' anxiety-depression-sleep disturbance symptom network, and make it difficult for students to have good sleep quality. Several studies have shown that pharmacotherapy, behavior therapy, or simple expressive writing can effectively mitigate daily anxiety (56, 58). Additionally, the “Moving to Emptiness Therapy Technique” based on Chinese culture has also shown excellent treatment effects for mental health problems (59). These methods should be considered in psychological intervention programs for high school students.

We also found a strong correlation between “Difficulty maintaining sleep” and “Early morning awakening” (YSIS4–YSIS5). This result matches the actual daily routine of Chinese high school students. Most Chinese high school students' age range is 13–18, a period when physical and mental development is susceptible to poor sleep quality (60). However, 75.24% of Chinese adolescent students over 13 years old can not reach the recommended 8 h of sleep (61). According to Zhang's study (23), on average, Chinese high school students spend more than 9.8 h learning at school and more than 3 h finishing homework, making more than 65% of students have to get up before 6:30 am and go to bed after 11:30 pm. Sleeping late and rising early reflects the true routine of Chinese students for heavy academic burden. Therefore, effective actions should be taken to reduce students' academic burden and ensure enough and good sleep quality for them.

The network comparison results showed that networks between junior and senior high school students did not differ significantly in edge weights, indicating that the two groups have similar network structures. However, the sum of all edge weights in senior high school students was greater than in junior high school students. This result revealed that the internal association between anxiety, depression, and sleep disturbance symptoms in senior high school students is tighter and stronger. In other words, the three symptom clusters from a more severe self-reinforcing feedback loop among senior high school students. Activating any single symptom causes a more significant change in the entire network. This phenomenon may be due to more serious mental health problems. Senior high school students face more academic pressure, conflicts, and more frequent depression and anxiety moods (34). Recurrence and longer-lasting negative emotions cause different symptoms to be activated more intensely and repeatedly, which leads to a denser network. Gijzen (62) also reported an approximate case that adults have a denser depression symptom network than adolescents for resurgence and long-lasting negative emotions.

The results about bridge symptoms also reflected students' psychopathological development process. Anxiety symptom “Nervousness” (GAD1) was the bridge node in the junior high group, while in the senior high group, depression symptom “Sad mood” (PHQ2) was the bridge node. This result suggested that anxiety is the key factor linking different disorders at the junior high stage, while at the senior high stage, it shifts to depression. During adolescent development, anxiety is always almost the primary condition for secondary depression (63), which means that in the early stage, adolescents often exhibit symptoms of anxiety caused by some external stressful event accompanied by mild depression symptoms. Whereas, under the long-term influence, students with mental problems develop an internal spontaneous depression pattern that is not affected by external events (64). This finding enlightens us that junior high school may be the generating stage of depression, anxiety, and sleep disturbances. However, in China, mental health resources in the junior high stage are pretty scarce (11). Chinese communities, schools, and experts should deploy more resources in psychological support and intervention for junior high school students in the future.

## Limitations

Some limitations should be mentioned. First, the current sample was selected from the general population. Thus, further testing requires further testing to determine whether the conclusions drawn from this study can be generalized to clinical samples. Second, although we used the network approach to explore the interwoven association among different symptoms across three syndromes, the cross-sectional research design cannot explore dynamic changes and causality. Further longitudinal extensions should be conducted. Third, since the aim is to obtain some enlightening results, the current study used a small number of items. Future studies could include more items, such as PHQ-9 and GAD-7, to explore the further relationship between different symptoms. Forth, sleep duration may affect depression, anxiety, and sleep disturbance symptoms, but we did not investigate relevant information. Future studies should incorporate sleep duration time into network analysis.

## Conclusions

In conclusion, this study is the first to analyze the depression-anxiety-sleep disturbance symptom network of Chinese high school students. We found that the core of high school students' sleep disturbance is difficulty falling asleep and waking up too early, which is closely linked to their enormous learning burden. Recently, the Chinese government has implemented a “double reduction” policy intended to reduce students' study burden (65). Whether this strategy can effectively mitigate sleep disturbance remains to be tested by future studies. We also explored differences in the network structures between junior and senior high school students and found the bridge node's transition from anxiety to depression. The results contribute to our understanding of adolescents' depression-anxiety courses and can assist us in identifying highly susceptible students and taking timely interventions to help them.

## Data availability statement

The raw data supporting the conclusions of this article will be made available by the author, without undue reservation.

## Ethics statement

The studies involving human participants were reviewed and approved by Beijing Normal University (Reference number: 202112220084). Written informed consent to participate in this study was provided by the participants' legal guardian/next of kin.

## Author contributions

SW took the lead in writing the manuscript. LZ and XL conceived the study design and supervised the data collection. YT and ZM performed the data analysis. WH, KL, YW, and ZX provided critical feedback and helped shape the research, analysis, and manuscript. All authors contributed to the article and approved the submitted version.

## Conflict of interest

The authors declare that the research was conducted in the absence of any commercial or financial relationships that could be construed as a potential conflict of interest.

## Publisher's note

All claims expressed in this article are solely those of the authors and do not necessarily represent those of their affiliated organizations, or those of the publisher, the editors and the reviewers. Any product that may be evaluated in this article, or claim that may be made by its manufacturer, is not guaranteed or endorsed by the publisher.
