# Supplementary material for: Mapping network connection among symptoms of anxiety, depression, and sleep disturbance in Chinese high school students
Source: Front Public Health. 2022 Sep 23;10:1015166. doi: 10.3389/fpubh.2022.1015166 (PMC9710521; doi:10.3389/fpubh.2022.1015166)
Supplement: Supplementary file 1 [file Data_Sheet_1.docx]

**Table S1.** Weighted adjacency matrix of PHQ-2, GAD-2 and YSIS-3 item scores (all students).

|  | PHQ1 | PHQ2 | GAD1 | GAD2 | YSIS3 | YSIS4 | YSIS5 |
| --- | --- | --- | --- | --- | --- | --- | --- |
| PHQ1 | 0 | 0.408 | 0.159 | 0.076 | 0.123 | 0.020 | 0.013 |
| PHQ2 | 0.408 | 0 | 0.180 | 0.183 | 0.059 | 0.049 | 0.035 |
| GAD1 | 0.159 | 0.180 | 0 | 0.566 | 0.081 | 0.037 | 0.018 |
| GAD2 | 0.076 | 0.183 | 0.566 | 0 | 0.055 | 0.035 | 0.077 |
| YSIS3 | 0.123 | 0.059 | 0.081 | 0.055 | 0 | 0.247 | 0.267 |
| YSIS4 | 0.020 | 0.049 | 0.037 | 0.035 | 0.247 | 0 | 0.460 |
| YSIS5 | 0.013 | 0.035 | 0.018 | 0.077 | 0.267 | 0.460 | 0 |

**Table S2.** Weighted adjacency matrix of PHQ-2, GAD-2 and YSIS-3 item scores (junior high school students).

|  | PHQ1 | PHQ2 | GAD1 | GAD2 | YSIS3 | YSIS4 | YSIS5 |
| --- | --- | --- | --- | --- | --- | --- | --- |
| PHQ1 | 0 | 0.399 | 0.146 | 0.082 | 0.108 | 0.028 | 0.023 |
| PHQ2 | 0.399 | 0 | 0.180 | 0.165 | 0.082 | 0.029 | 0.029 |
| GAD1 | 0.146 | 0.180 | 0 | 0.528 | 0.088 | 0.055 | 0.030 |
| GAD2 | 0.082 | 0.165 | 0.528 | 0 | 0.046 | 0.039 | 0.103 |
| YSIS3 | 0.108 | 0.082 | 0.088 | 0.046 | 0 | 0.261 | 0.262 |
| YSIS4 | 0.028 | 0.029 | 0.055 | 0.039 | 0.261 | 0 | 0.440 |
| YSIS5 | 0.023 | 0.029 | 0.030 | 0.103 | 0.262 | 0.440 | 0 |

**Table S3.** Weighted adjacency matrix of PHQ-2, GAD-2 and YSIS-3 item scores (senior high school students).

|  | PHQ1 | PHQ2 | GAD1 | GAD2 | YSIS3 | YSIS4 | YSIS5 |
| --- | --- | --- | --- | --- | --- | --- | --- |
| PHQ1 | 0 | 0.415 | 0.162 | 0.068 | 0.132 | 0.016 | 0 |
| PHQ2 | 0.415 | 0 | 0.179 | 0.196 | 0.041 | 0.066 | 0.040 |
| GAD1 | 0.162 | 0.179 | 0 | 0.582 | 0.073 | 0.027 | 0.013 |
| GAD2 | 0.068 | 0.196 | 0.582 | 0 | 0.061 | 0.035 | 0.063 |
| YSIS3 | 0.132 | 0.041 | 0.073 | 0.061 | 0 | 0.237 | 0.272 |
| YSIS4 | 0.016 | 0.066 | 0.027 | 0.035 | 0.237 | 0 | 0.471 |
| YSIS5 | 0 | 0.040 | 0.013 | 0.063 | 0.272 | 0.471 | 0 |

**Table S4.** Means, standard deviations, *t*-test *p*-value and *Cohen's d* in junior high school students (N = 13999) and senior high school students (N = 12550).

|  | **Junior high (*N=***13999***)*** | **Senior high (*N=***12550***)*** | ***p*** | ***Cohen’s d*** |
| --- | --- | --- | --- | --- |
| PHQ1 | 0.38 (0.73) | 0.62 (0.90) | <0.001 | 0.302 |
| PHQ2 | 0.29 (0.65) | 0.48 (0.80) | <0.001 | 0.271 |
| GAD1 | 0.28 (0.63) | 0.53 (0.85) | <0.001 | 0.334 |
| GAD2 | 0.21 (0.59) | 0.43 (0.82) | <0.001 | 0.301 |
| YSIS3 | 1.44 (0.91) | 1.64 (1.12) | <0.001 | 0.201 |
| YSIS4 | 1.33 (0.79) | 1.41 (0.92) | <0.001 | 0.095 |
| YSIS5 | 1.30 (0.79) | 1.38 (0.92) | <0.001 | 0.101 |

**
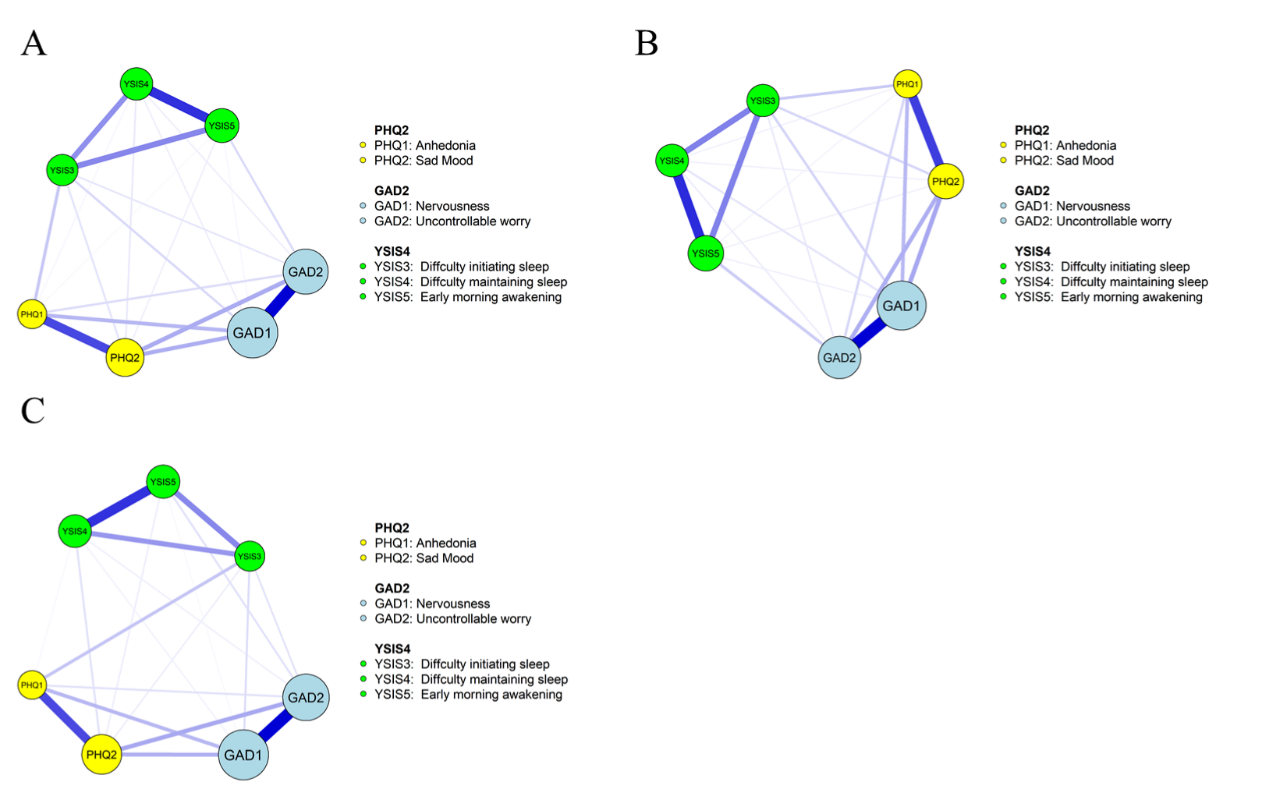
**

**Figure S1.** The original network structures. A, network of all students. B, network of junior high school students. C, network of senior high school students.

**
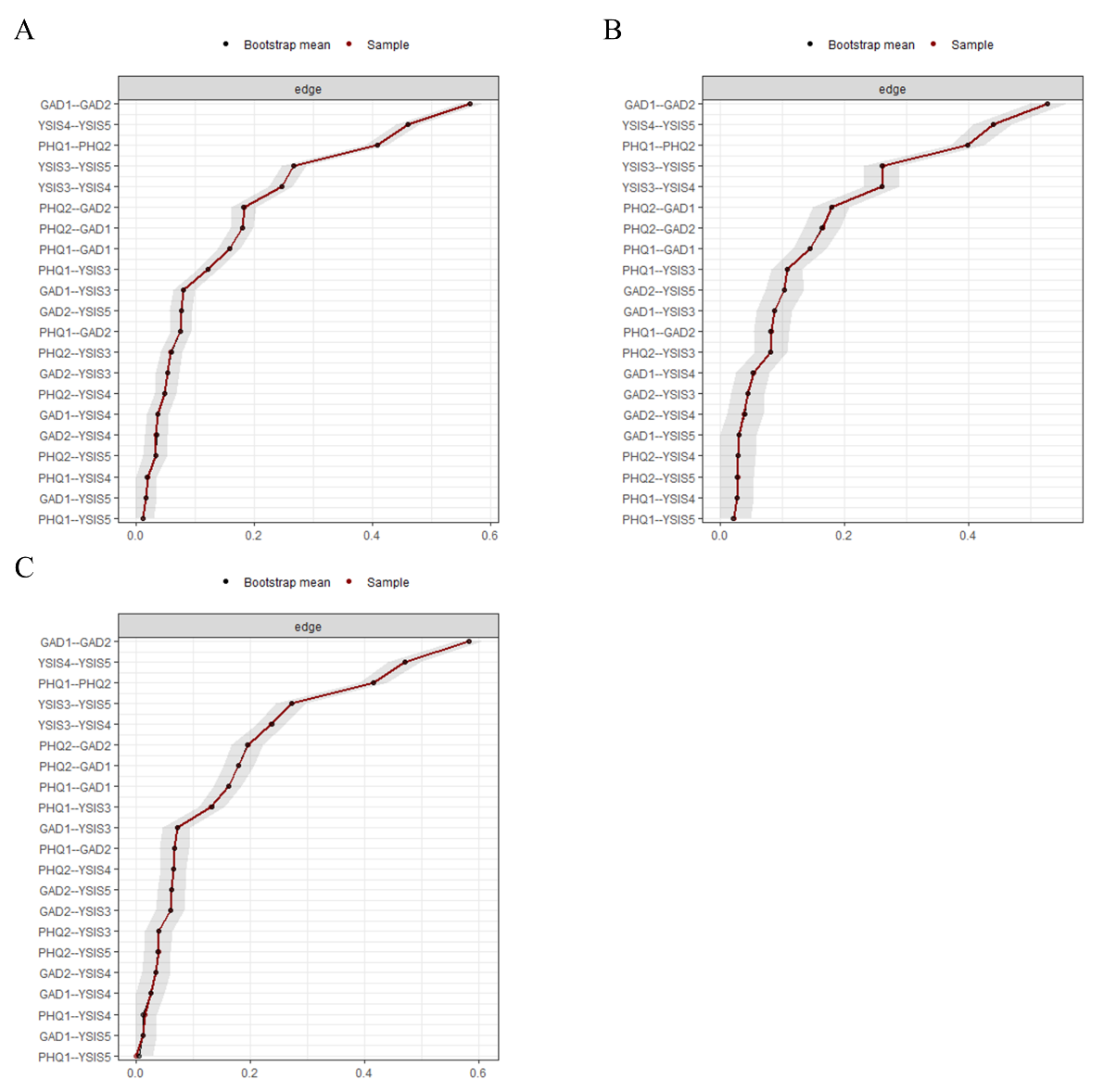
Figure S2.** Nonparametric bootstrapped confidence intervals of estimated edges. The

red line represents the estimated edge, while the dark area indicates the 95% bootstrap

confidence interval. A, all students. B, junior high school students. C, senior high school students.

**
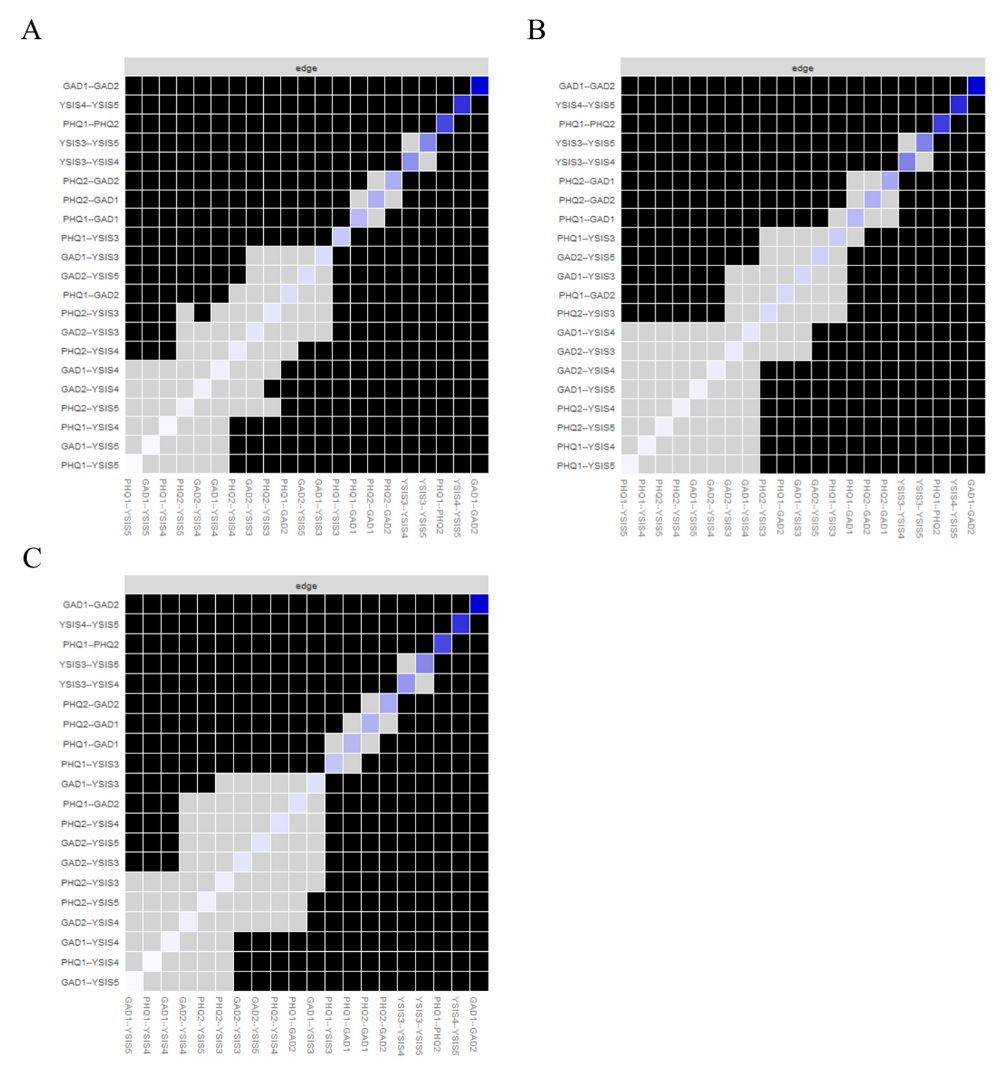
Figure S3.** Bootstrapped stability test for edge-weight. The results of the bootstrapped

difference tests (*α* = 0.05) for edge-weights were shown in this figure. The color of the boxes indicates whether edge-weights differ significantly from each other (i.e., black) or do not differ significantly (i.e., grey). The diagonal line indicates the strength of edge-weights, shifting from red (negative associations) to white (representing weaker edges) and ultimately blue (representing stronger edge-weights). A, all students. B, junior high school students. C, senior high school students.

**
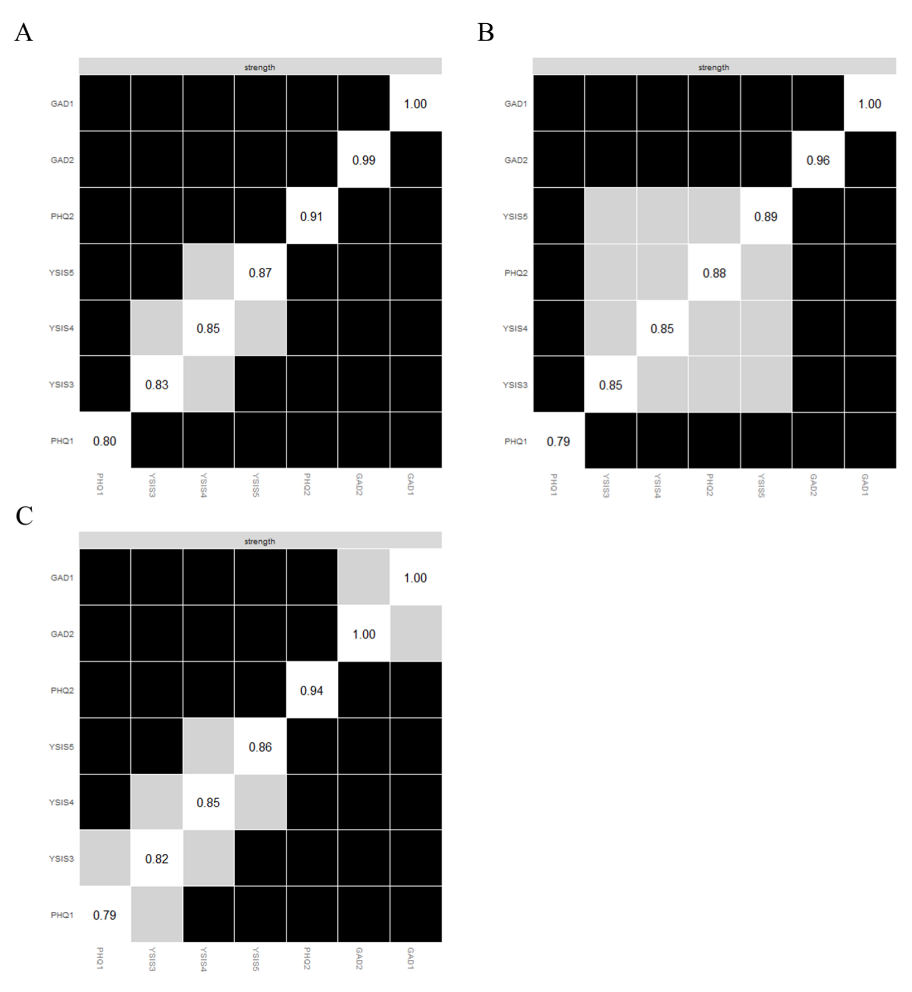
Figure S4.** Nonparametric bootstrapped difference test for nodes. Grey boxes indicate

no significant difference, whereas black boxes indicate a statistically significant difference (*p* < 0.05). Diagonal color and saturation represent the magnitude and direction of each estimated edge. A, all students. B, junior high school students. C, senior high school students.


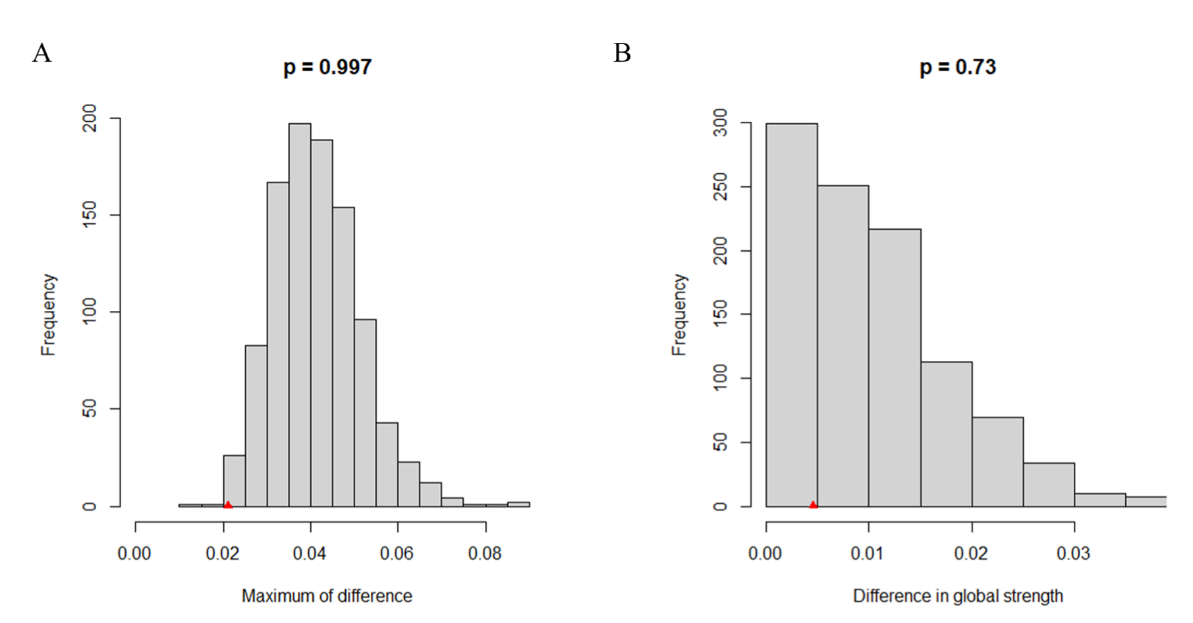


Figure S5. NCT results between the original network and the new network among all students. A, network edge invariance. B, network global invariance.


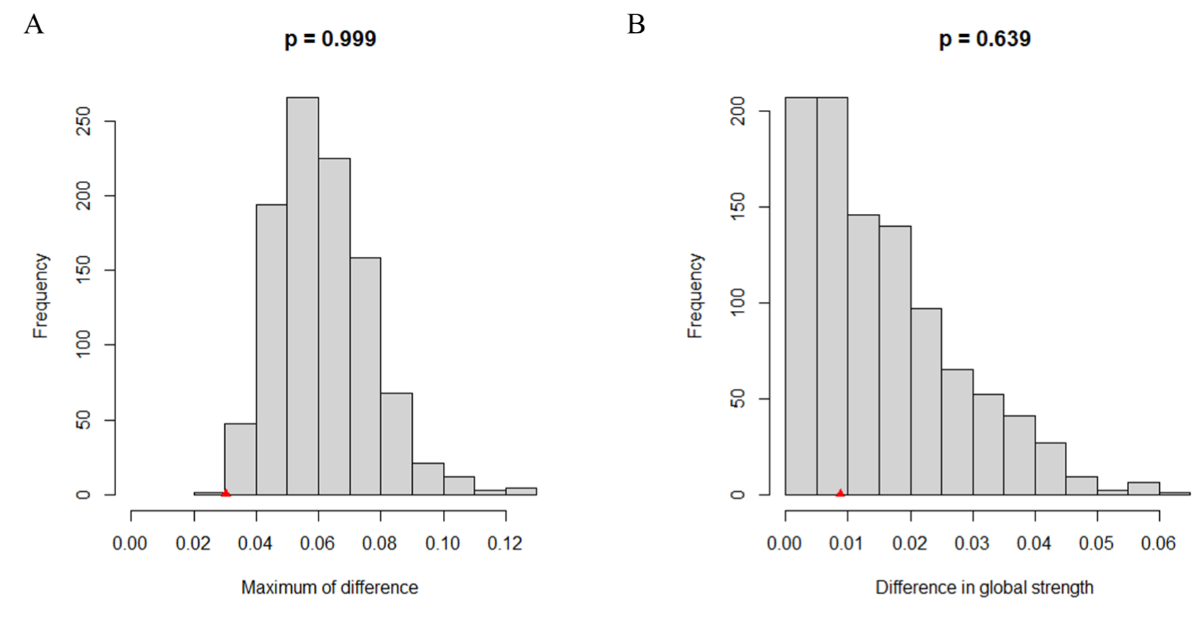


Figure S6. NCT results between the original network and the new network among junior high students. A, network edge invariance. B, network global invariance.


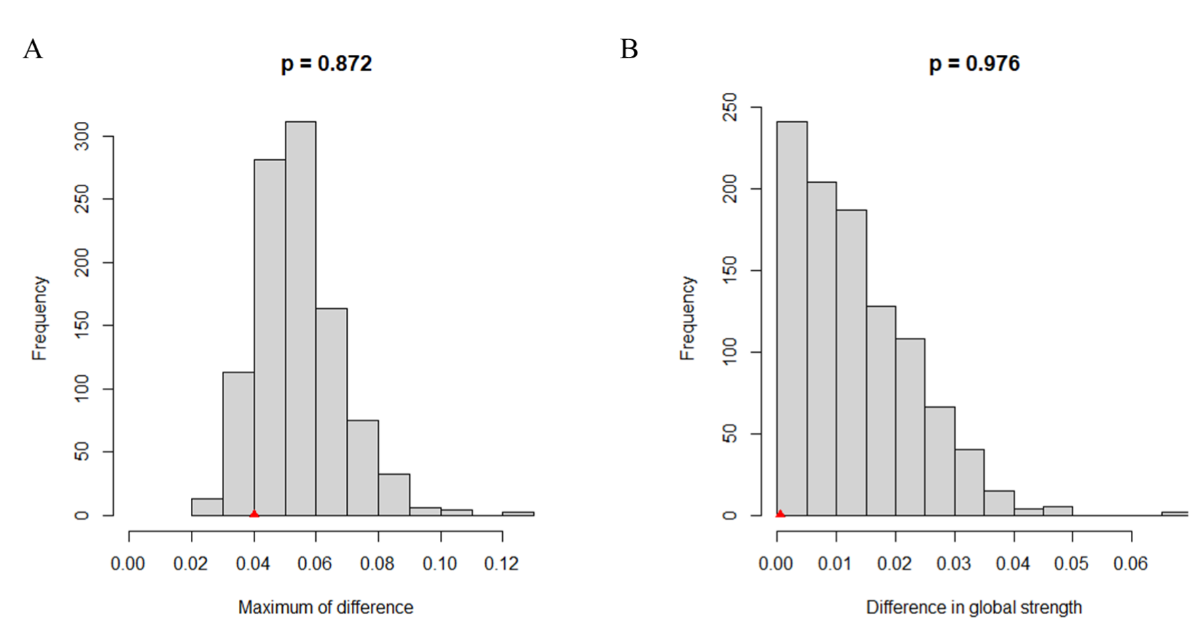


Figure S7. NCT results between the original network and the new network among senior high students. A, network edge invariance. B, network global invariance.
